# Agile User-Centered Design of a Clinical Research Project Management System in a Pediatric Health Institute

**DOI:** 10.1055/a-2625-1046

**Published:** 2025-07-09

**Authors:** Danny T.Y. Wu, Manhar Suharwardy, Aniruddhan Ramesh, Shubhra Gupta, Chen Xin, Ting Zhang, Ching-Tzu Tsai, Jenna Dawn, Michelle Faust, Adam Kushner

**Affiliations:** 1 Department of Biostatistics, Health Informatics and Data Sciences, University of Cincinnati College of Medicine, Ohio, United States; 2 Department of Pediatrics, University of Cincinnati College of Medicine, Cincinnati, Ohio, United States; 3 Medical Sciences Baccalaureate Program, University of Cincinnati College of Medicine, Ohio, United States; 4 School of Design, College of Design, Architecture, Art, and Planning, University of Cincinnati, Ohio, United States; 5 Department of Computer Science, College of Engineering and Applied Sciences, University of Cincinnati, Ohio, United States; 6 Heart Institute, Cincinnati Children's Hospital Medical Center, Cincinnati, Ohio, United States; 7 School of Information and Library Science, University of North Carolina at Chapel Hill, North Carolina, United States

**Keywords:** research planning and conduct, interfaces and usability, workflow, evaluation, user acceptance and resistance

## Abstract

**Background:**

Project management is crucial in academic hospitals due to the intensive involvement in research and clinical trials. Designing an application in a user-centered and workflow-compatible manner can prevent issues arising from hospital's limited resources and various risks (e.g., financial risk and ethical misconduct).

**Objectives:**

This study aimed to (1) develop a hybrid method combining user-centered design (UCD) and agile software development (ASD) principles, (2) apply the hybrid model to develop a Clinical Research Project Management System (CRPMS), (3) assess the usability of the CRPMS and iteratively refine the application.

**Materials and Methods:**

A CRPMS was developed following the UCD and ASD principles and supported by a research core in a pediatric heart institute. In Phase 0, semi-structured interviews were conducted to understand processes and bottlenecks. In Phase 1, the project management and budgeting sections of the CRPMS were simultaneously developed. In Phase 2, the Principal Investigator dashboard and the intake section of the CRPMS were developed. Usability evaluation metrics included the System Usability Scale (SUS), Single Ease Question, and severity scores.

**Results:**

In Phase 1, the average SUS score was 88.65. There were 126 usability issues and 68 were considered high severity due to the SUS score cutoff (>1.5). In Phase 2, the average SUS score was 87.1. All of the 71 usability issues were addressed during the iterative refinements.

**Conclusion:**

A workflow-compatible and highly user-friendly CRPMS was developed by employing a hybrid Agile UCD model. Future work includes continuing development and expanding the CRPMS within and outside the institute.

## Background and Significance

Project management is a mature discipline that employs a systemic and effective approach in planning and executing projects. 1 It provides a clear definition of the roles and responsibilities of all project participants to clarify the expectations for everyone involved. 2 Project management reduces the risk of delays and loss of progress by providing a framework to execute the project. 2 In most cases, projects tend to overrun the time frame or budget, leading to unfavorable outcomes. 3 Effective project management ensures that projects are delivered on time, within budget, and to the satisfaction of stakeholders, contributing to the overall success of organizations. 4

In project management, there are generally five key processes to follow throughout as a project's life cycle: (1) initiation, (2) planning, (3) execution, (4) monitoring and controlling, and (5) closure. 5 Additionally, there are nine knowledge areas that cover various aspects of project management, including (1) integration, (2) scope, (3) time, (4) cost, (5) quality, (6) resources, (7) communication, (8) risk, and (9) procurement. 5 Together these form a structured framework that project managers can adapt and tailor to meet the unique requirements of their specific projects and organizations. By understanding and applying these principles, project managers can effectively plan, execute, and deliver projects while ensuring project success and stakeholder satisfaction. 5

Academic hospitals serve as centers of extensive research and clinical trials making it crucial to practice research project management. 6 The research conducted by such hospitals is very complex, expensive, high stakes, and must be completed with limited resources and funding. 1 Therefore, there is a risk of delivering poor results and causing project failure. Hospitals have been addressing these challenges by applying project management principles, as it enables effective resource management, sets reasonable objectives and milestones, and monitors progress ensuring the completion of a project within the limited resources. 2

Research project management can be complex and multilayered therefore implementing a project management application to support the whole process can be challenging. 1 2 While there are several commercial applications made available to support clinical trial management, there is a noticeable lack of applications that can improve the awareness of clinical research staff while designed to be workflow-compatible and highly usable. Workflow compatibility and perceived ease of use are critical because they directly impact the user satisfaction and technical adoption of an application. 7 8 User input therefore is essential to develop such applications to minimize the gap in mental models between the designers and the target users. 9 10

In this project, we developed a clinical research project management system (CRPMS) in a user-centered and workflow-compatible manner. Specifically, this study aimed to (1) develop a hybrid method combining user-centered design (UCD) and agile software development (ASD) principles, (2) apply the hybrid model to develop a CRPMS, (3) assess the usability of the CRPMS and iteratively refine the application.

## Materials and Methods

### Overview of User-Centered Design and Agile Software Development

UCD and ASD are two complementary methodologies that emphasize meeting user needs and creating high-fidelity prototypes. UCD is a human-centric cyclical process that involves four distinct phases: understanding the user context, specifying user requirements, designing solutions, and conducting evaluations. 11 12 Central to the UCD methodology is usability testing, where real or proxy users engage with prototypes and perform a series of designated and realistic tasks. 10 13 14 This process allows designers to observe user interactions, identify potential issues, pain points, and areas requiring enhancement to improve the prototype. 12 15 The insights gained from usability testing become pivotal in driving iterative design decisions and implementing improvements to optimize user satisfaction. 16 17 Subsequent interviews or surveys with users are often conducted, providing designers with valuable data on user preferences and unmet needs. 16 18 19

ASD, on the other hand, is a flexible and iterative approach, which emphasizes incremental development, enabling teams to deliver working software in short and frequent cycles called “sprints.” 20 During each sprint a specific set of functionalities is developed, tested, and delivered to stakeholders/users. 20 Agile teams continuously gather feedback and insights from stakeholders, including end-users, and adapt the software accordingly. 21 22 Moreover, the feedback-driven design approach promotes iterative refinement and adaptation to better meet users' evolving expectations resulting in a prototype that provides the highest user satisfaction. 21 22 For example, Scrum is an ASD that divides the project into sprints of 2 to 4 weeks. 23 24 Each sprint involves planning, executing, review, and future improvement phases. 23 24 A scrum team usually includes a product owner, a scrum master, and the development team. 23

### A Hybrid Model—Agile User-Centered Design

Based on our literature search on PubMed to determine the use of combining UCD and ASD, it was found that only six studies adopted such methodology. However, the objective of these studies is to create mobile/web applications that cater to specific health issues rather than project management in a clinical setting. 25 26 27 28 29 30 There were three commonly used methods from these papers: usability testing, semi-structured interviews, and iterative improvements of the prototype. 25 26 27 28 29 30 For example, the HealthPAL study's UCD process involved multiple rounds of usability testing, where feedback from participants informed the development of subsequent prototypes. 30 This was in parallel to ASD where a team of designers collaborated to transform user requirements into actionable software features followed by iterative rounds of continuous improvement resulting in a high-fidelity prototype.

While this hybrid methodology is recently being used more often in clinical and health informatics, it has been researched in computer science and software engineering field since ASD and UCD have overlapped concepts and unique benefits in developing software applications. Agile UCD is one such hybrid methodology. A systematic review of Agile UCD identified best practices for combining agile methodologies with user-centered designs to create highly successful applications. 31 Our study employed a modified Scrum methodology, which is one of the most prevalent agile methodologies used in combination with UCD techniques to create this hybrid model. 31 UCD is relatively more time-consuming compared with the fast-paced ASD methodologies. 31 To effectively combine UCD and agile methodologies, our study placed the time-consuming UCD components, such as need assessment and user-centered evaluation, at the beginning and end of each sprint, respectively.

**Fig. 1:**
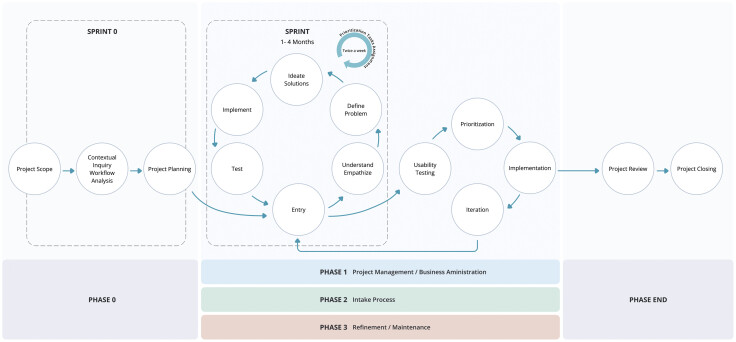
Agile UCD study design. UCD, user-centered design.

This allowed us to integrate the core principles of UCD with the agile framework. Additionally, we modified the Scrum process to meet weekly instead of daily, accommodating the capacity of both developers and designers. The scrum team was modified to include users and a joint team of designers and developers. Our study used Agile UCD hybrid methodology ( Fig. 1 ). The same method has been adapted in our previous work.

### Study Setting

This case study was conducted in a heart institute research core (HIRC) 32 at a leading nonprofit children's hospital. As one of the nation's top institutions for pediatric scientific discovery and advancement, this research core actively engages in clinical trials and translational research across a wide range of fields. HIRC stays at the forefront of advancements in pediatric cardiology, ensuring that patients have access to the most advanced diagnostic tools and treatment options. 32 All of the above highlight the need for a CPRMS.

### Study Design

The study was conducted in three phases as illustrated in Fig. 1 . In Phase 0, semi-structured interviews were conducted with the participants to understand the current processes and identify inefficiencies and areas for improvement. In Phase 1, designers concurrently developed the project management and budget components of the CRPMS. This was an iterative process with the development of low-fidelity mockups by the designer of the research team based on user feedback from each round of usability testing sessions. Our methodology incorporated a specific model of teamwork, referred as “Dev-Design Synergy,” during development of the prototypes. 33 In this model, the designers led usability testing sessions and collected user feedback on prototypes, with the developers participating in such sessions. Dev-Design Synergy 33 is crucial to ensure the fulfillment of the design and functionality requirements of a technical solution. Phase 2 adhered to the same development cycle to create an intake section. In both Phase 1 and 2, user feedback was collected via task completion, System Usability Scale survey (SUS, as defined in Supplementary Appendix 1 ), and Single Ease Question (SEQ, details in the Usability Testing section). Subsequently, identified issues were presented to developers in an internal team meeting for resolution, and the designers and developers used the same tool (ClickUp) to track issues and communicate system requirements. These shared tools and intertwined processes led to increased and harmonious collaboration between the designers and developers ensuring that both Agile and UCD methodologies were equally prioritized in the creation of the application. Lastly, the cycles of continuous refinement and testing were referred to as Phase 3 in Fig. 1 .

### Participant Recruitment

The participants were either staff members focused on business or clinical research operations, or the faculty members and principal investigators (PI) who made requests for research support in the study site. Recruitment was conducted using convenience sampling through the professional network of the research team and the HIRC lead operations and business managers via email invitations. For Phase 0 and 1, the team consisted of project managers, clinical research coordinators, and regulatory specialists. Members of this team provided information on the day-to-day logistics, planning, and regulatory compliance related to research activities. For Phase 2, the participants were PIs who were both current and potential users of the CPRMS. Due to the small number of total participants, demographic information was not collected to protect privacy as approved by our IRB. In sum, 9, 12, 7 participants were recruited in Phase 0, 1, 2, respectively.

### Usability Testing Session

**Fig. 2:**
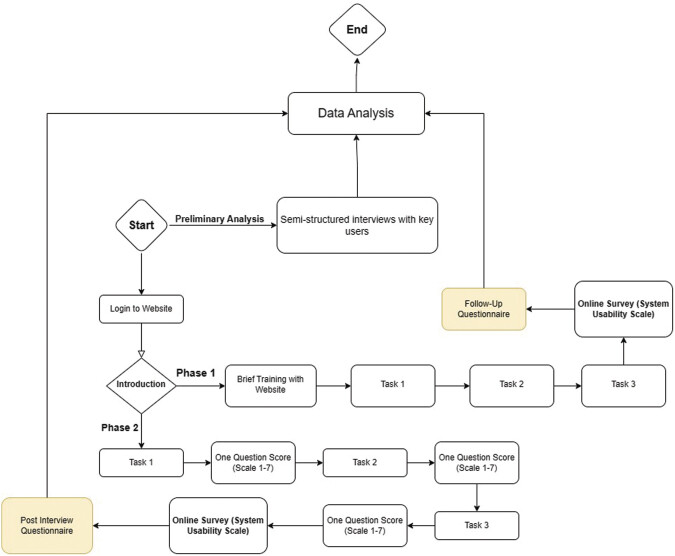
Usability testing.

To assess the prototype's user-friendliness, 2 usability testing was performed through one-on-one sessions lasting 30 to 45 minutes. The process of usability testing sessions can be seen in Fig. 2 , where the usability of the system was assessed by the SUS (subjective measure) and by the observations of the research team. During the observation, the research team recorded the correctness of each task (accuracy) and how long each task was completed (efficiency). The usability testing sessions were conducted in Phase 1 and 2.

In Phase 1, the user interface consisted of two pages: Project List and Single Dashboard. Each usability testing session began with the moderator introducing the system to the participants and providing an overview of the intended functions to facilitate their understanding of the system. Then using think-aloud strategy participants were given workflow-relevant, role-based tasks to complete. These tasks were team-dependent and realistic (e.g., select a project, go to initiate and plan phase, and enter data) and codesigned by the research team and the HIRC managers. After task completion, participants filled out the SUS survey, one of the most frequently used questionnaires to measure usability of a system and shared any improvements/comments regarding the interface. The SUS survey consisted of 10 questions on a 5-point Likert scale. After the scoring process, each participating user's composite score was calculated from 0 to 100. A score of 68 or higher indicated “good” usability, whereas a score of fewer than 68 indicated “poor usability.” 34 Lastly, there were follow-up questions asked by the facilitator to clarify any confusion that might have occurred during the sessions.

In Phase 2, each session began with the participants logging into the HIRC website with their CCHMC accounts. Participants were then given three scenario-based tasks to complete associated with their typical workflow. The tasks were created to assess the design features such as submitting intake requests and checking project status. Participants employed think-aloud strategy as they completed the tasks. After the completion of each task, an SEQ was used to assess the ease in task completion on a scale from 1 to 7. Participants were asked to take the SUS survey after the completion of all tasks in the same manner as Phase 1. Each session ended with post-interview questions asked by the facilitators to clarify any confusion regarding the functionality of newly implemented features and get improvement feedback.

### Data Collection and Analysis

**Fig. 3:**
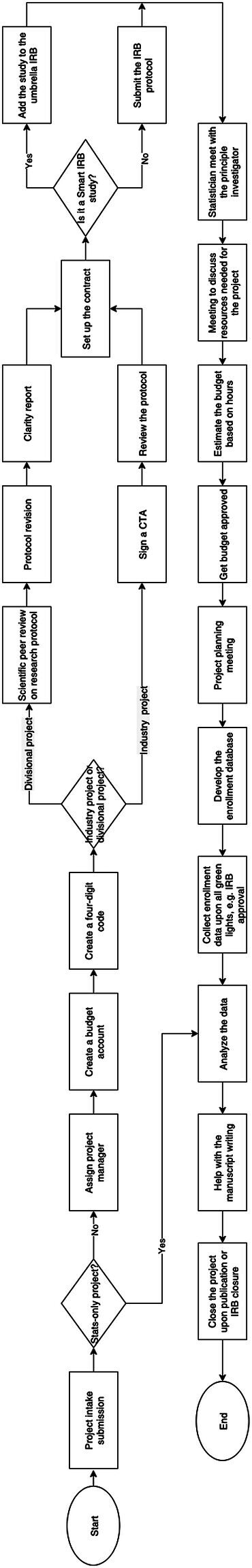
Consolidated HIRC workflow. HIRC, heart institute research core.

As mentioned above, semi-structured interviews were conducted in Phase 0, which included five topics: (1) job title and responsibility, (2) daily process work-through, (3) workflow issues and bottlenecks, (4) potential solutions to these issues, and (5) the role and functionality of the dashboard. Follow-up questions were asked based on the participants' responses. These interviews were recorded and transcribed verbatim. The research team reviewed the interview data to generate a workflow diagram for each participant. Then, the individual workflow diagrams were merged based on the teams and then consolidated as one high-level diagram to illustrate the key stages ( Fig. 3 ). In addition, a thematic analysis was conducted following the six-step guideline 35 to identify and categorize key issues (pain points). This process involved reviewing the data, coding the relevant information, and grouping the codes into clear themes ( Table 1 ). This analysis aimed to highlight the main pain points, such as unclear content, by organizing them into distinct categories based on the recurring patterns observed across the data.

**Table 1:** Pain points in the current workflow of the research project management

Pain points	Definition	User's comments
Low usability of current tools	How easy the current tools are to use	“It's an incredibly manual process”
Redundancy and inefficiency	Redundancy occurs when the same piece of data exists in multiple places. And inefficiency when the same data exists in different formats in multiple tables/excels	“Maintain data in multiple places and then put it in multiple places,” “it's not easy for the project managers to access all that information at one time because they're all individual excels”
Poor data quality	Inaccurate or incomplete data or outdated information	“Things wasn't getting recorded,” “Things could get lost”
Low information availability	The information is not readily available to authorized users	“A lot of back and forth asking,” “a lot of work [to] figure out”

In Phase 1, each usability testing session was video recorded. The research team reviewed these recordings to capture usability issues. In addition, the SUS scores were calculated and compared among the teams. Each of the usability issues was further scored in terms of impact, criticality, and frequency. Specifically, impact was recorded using Fibonacci-based scaling from 1 to 5 13 because this measure allows for more detailed differentiation between levels of impact, especially for issues that vary in severity. Criticality was recorded from 1 to 3. Frequency was measured by dividing the number of users with the number of times an issue was mentioned in each group of testing. These three aspects (Impact, Criticality, Frequency) were multiplied to form a severity score for the issue. 13 The issues were prioritized based on the severity score as defined in Supplementary Appendix 2 (sorted from large to small) and discussed with users in the weekly meetings to seek solutions. The supplement file provides details of the SUS and the severity score calculation.

In Phase 2, there was the addition of a SEQ to the usability evaluation metrics. The SEQ was worded as “Overall, how difficult or easy were the tasks to complete?” The SEQ was administered after each task was completed whereas the SUS was administered after the completion of all tasks. This score ranged from 1 to 7 where 1 meant the tasks were very difficult to complete and 7 meant they were very easy to complete. The post-interview questions served to evaluate the participants' level of comfort with the newly integrated features. There was not a severity score created as there was enough time to fix all the issues encountered during usability testing sessions.

## Results

### Workflow Analysis ( *N* = 9)

In Phase 0, the workflow analysis of the current system was performed, resulting in the generation of a consolidated workflow diagram ( Fig. 3 ). The workflow diagram consists of seven stages, including (1) intake, (2) initiate, (3) plan, (4) collect, (5) analyze, (6) end, and (7) track. Moreover, the workflow analysis also identified four major pain points in the current workflow, (1) low usability of current tools, (2) redundancy and inefficiency, (3) poor data quality, (4) low information availability ( Table 1 ). These pain points were evaluated and addressed during the mockup development.

### Usability Evaluation of Phase 1 ( *N* = 12)

In Phase 1, a total of 12 participants were recruited for the usability testing sessions. One of the regulatory team members did usability testing for both teams. The overall SUS score was 88.65 (>68), indicating the above-average usability of the prototype system ( Table 2 ). A total of 126 usability issues were reported and ranked based on the severity score. After reviewing the top usability issues, an arbitrary cutoff of severity score (>1.5) was determined to classify issues as high severity. This cutoff was determined arbitrarily based on the number of issues and the capacity of the development team. Using this cutoff, 68 usability issues (54%) were considered as high severity among a total of 126 issues ( Table 3 ). After group discussions with the leaders in the project management and business team, it was found that 15% ( *N* = 10) of the 68 high-severity issues required user training to bridge the mental model in system functionality. Of note, we used the data from the think-aloud protocol and the user comments as a reference but did not conduct qualitative analysis on them to focus on fixing the usability issues and preparing for Phase 2.

**Table 2:** Phase 1 System Usability Scale survey

Team\SUS scores	Number of users	Min	Max	Average	Standard deviation
Project management	4	90.00	92.50	91.25	1.7678
Business	4	75.00	87.50	82.50	7.0711
Regulatory	5	77.50	100.00	91.50	1.7678

Abbreviations: Max, maximum; min, minimum; SUS, System Usability Scale.

**Table 3:** Phase 1 summary of usability issues

Team\number of issues	Total Number of Issues	High severity issues (>1.5)	Requires user training
Project management	53	20	5
Business	34	21	1
Regulatory	39	27	4
Total	126	68 (54% of 126)	10 (15% of 68)

### Usability Evaluation of Phase 2 ( *N* = 7)

**Fig. 4:**
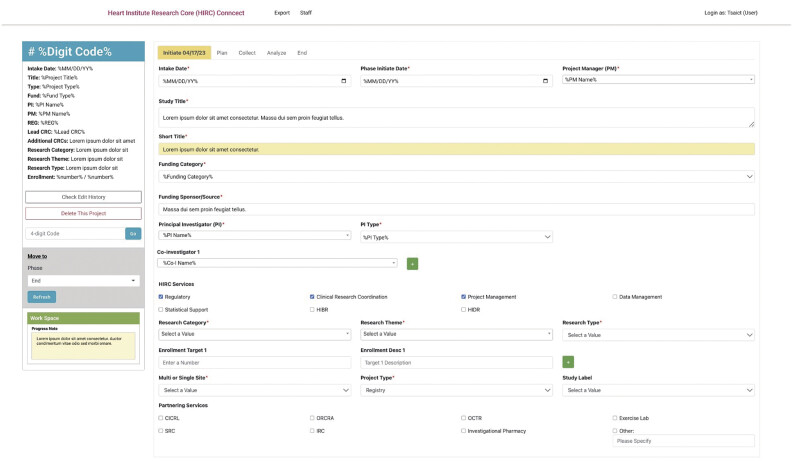
Revised prototype of the initiate phase of the Clinical Research Project Management System (CRPMS). Note: 04/17/23 is an arbitrary project initiation date.

In Phase 2, a total of seven participants were recruited for the usability testing sessions on the revised prototype ( Fig. 4 ). The transcript analysis of the usability testing sessions revealed a total of 71 usability issues. The thematic analysis identified four key issues shared by all seven users ( Table 4 ): (1) unclear content and options, (2) uncertainty about how to proceed without answers, (3) the need for more guidance and terminology support, and (4) a desire for additional features to track project status. All the issues were circled back to the HIRC lead operations and business managers to determine the improvement necessary for the application, because the system was more stable, and the development team had bandwidth to address all issues. The prototype system's overall SUS score was 87.1 (>68), indicating above-average usability.

**Table 4:** Phase 2 major issues/pain points

Theme/issues	Descriptions	Quotes
The content (question and options) is unclear to the users	The content needs to be reviewed and determined again because some of the terms are unclear to the users, users' answers might overlap between two options, or users' answers might look for other options to select	“You may wanna add that because of NIH. They're now requiring that data and specimens go to a central national repository, so you're gonna need to put that as an option here, because this is just hire be approved institutional. So, there needs to be one for National repository.”—P3 “I would love if there is special location services recruitment in the cardiology clinics would be nice or just outpatient area in general because we do a lot of recruiting there”—P7
Users were unsure of what to do when they didn't have answers	When users encountered a question couldn't answer, they often wondered if there was an option for “unsure.” Occasionally, users wished to leave certain fields blank. However, they were unable to proceed to the next page due to unanswered questions	“I think actually having like a check box next to a question that says does not apply to my study or something like that where you could just skip that question or anything to that question without actually filling anything in.”—P5 “And the old one it's been a while since I filled one out, but it seemed like there were some parts I had to fill in that may or may not have applied and there was more free texting of things as opposed to being given what the various options were, so it seemed easier to get through”—P6
Users require more guidance and knowledge to understand the terminology and answer the questions	When users are unfamiliar with the system's knowledge, they need guidance, definitions, or additional descriptions for each value, option, question, and even predetermined institutional	“I guess the question is when it's more than one of these causes the biorepository generally includes other ones that might be good to have a link here where you could click on each of these and then figure out what the definition of these things is and what they're supposed to do.”—P1 “It could have a brief definition like the definition of “End” means study completed.”—P2
Users are seeking for more functions to help them know more about their project status.	Users find the project status pill is not obvious. Therefore, they are looking for more abilities to manage their projects. For example, they seek streamlined notifications for project status updates and convenient access to documents, particularly from the IRB. Users also emphasize the importance of transparent timelines of project phases.	“I don't know if you needed some other like notification like accepted pending, you know, whatever assignment of staff or something like that's what I was looking for.”–P4 “Is it just submitted and accepted or it would be nice to sort of what the timeline of those steps is? We're kind of timeline that shows the stages and highlights where your project sits in*–P5

## Discussion

### Key Findings

In this study, we developed a hybrid model combining UCD and ASD, applied the hybrid model to develop a CRPMS, and assessed the usability of the CRPMS to iteratively refine the application. We combined a modified scrum methodology with UCD to create our Agile UCD model, which allowed for the iterative development of the CRPMS and ensured that user feedback was incorporated throughout the development process. The application's high usability indicates the success in our hybrid method, and the feedback-driven iterative improvement that bridged the gap in the mental models between the users and the designers. It is worth noting that the methodology of usability testing in Phase 2 was adjusted slightly to include the SEQ after each task to obtain more feedback from the users. Ultimately, both phases had high usability scores and identified critical usability issues for improvement. Our experience indicated that usability testing is not a one-size-fits-all methodology and should be adjusted based on the context and goals.

### Implications

#### Success of Agile User-Centered Design in Clinical Research Informatics

There are past research studies utilizing the hybrid model; however, this is the first study to show the success of the Agile UCD model in the context of clinical research. Our hybrid model shows that it is feasible and effective to conduct iterative rounds of usability testing and refinements, although careful planning and leadership support were necessary to fit the UCD activities into the busy schedule of clinicians and clinical research staff. 36 The combination of Agile UCD model provides the advantages of both approaches, improving software usability and user experience. 37 Agile enables usability testing on working software and allows detecting and correcting usability issues later during the iteration process. 36 38 39 This leads to faster overall development time as there are less issues encountered post-implementation of applications. 37 38 40 The hybrid approach shows enhancements in the development process, including decreased rework, improved user satisfaction, and enhanced collaboration with stakeholders. 39 40 It also leads to a deeper understanding of users and their requirements.

#### Current Practices and Improvements

Usability testing is a critical aspect of application development because it minimizes the gap between the mental model of the designer and the user. 41 By tailoring the application to the users' needs and workflow, usability testing increases user retention and decreases workflow incompatibility. 17 41 Usability testing conducted in the past focused on quantitative measures, especially surveys, during the development and implementation of clinical decision support systems. 42 With the significant increase in the use of (mobile) health applications, 43 the usability testing methods have evolved to include both quantitative and qualitative measures, such as user interviews, observations, eye-tracking, and think-aloud strategy. 27 28 30 44 45 However, in the context of clinical research informatics (CRI) applications, usability testing remains limited. 42 Our study highlighted a need to create a culture of usability in the CRI area. By incorporating Agile UCD and conducting mixed-method studies to utilize the strength of qualitative and quantitative methods, applications can be more intuitive, efficient, and user-friendly. 41 46

#### Challenges of Agile User-Centered Design

A systematic literature review of past studies utilizing Agile UCD identified the most prevalent challenges associated with this hybrid model. 31 First challenge identified was “time constraints” in understanding user requirements due to Agile's fast development process. This was followed by issues maintaining workload balance between designers and developers, which led to the subsequent issue of prioritization among them. In our modified model, outlined in the methods section, the integration of both Agile and UCD methodologies was reinforced through harmonious collaboration and the Dev-Design Synergy. 33 This ensured that user feedback directly influenced the application's development, fostering effective problem resolution and stronger teamwork. Another challenge was that the user feedback might not include usability concerns for different groups. To address this, we conducted a workflow analysis in Phase 0 to comprehend the processes of research project management and included participants representing the target users for each part of the application. Additionally, we held weekly meetings and maintained email correspondence with users to gather their input on every usability concern that arose during development. In our hybrid model, we also addressed the challenge of documenting scenarios, stories, and tasks during development by utilizing digital tools including ClickUp and Figma. Both tools allowed us to track all aspects of development, including deliverables and methods of execution. Lastly, the challenges associated with “testing” were addressed by having three levels of testing before we marked an issue as resolved. The first level involved fixing the function and having it tested by other developers. The second level involved the designers testing the function associated with the usability issue. The third level involved the designers presenting the function to the users and having it approved. If at any point, the function was not cleared by the role next in line, it would move back to the initial development phase to go through testing again.

### Limitations

This study has three limitations. First, the CRPMS was specifically designed and developed to meet the needs of a single group in an institution. To determine the generalizability of the prototype system, more studies should be conducted in other clinical research settings. Second, the usability testing included 19 participants in two phases recruited through a convenience sampling. By including more participants, we may uncover more usability issues, ultimately resulting in a more refined final prototype. However, according to “the five-user rule,” when conducting usability evaluation five participants are often sufficient to uncover approximately 80% of usability issues. 47 This number of participants, even in various groups, should help us uncover enough key usability issues. Third, we did not employ other data collection methods, such as eye-tracking, nor a pre-post study design, to understand the nuance of user behaviors. Since the usability testing was conducted in tandem with project development, it was not practical to conduct a pre-post study. However, the current usability testing included think-aloud, realistic tasks, one-question and SUS surveys, and post-interviews, which should help us collect detailed user feedback. Next, the think-aloud method may slow down the usability testing. However, it provided valuable qualitative data on the participants' decision-making and thought processes. Lastly, cybersecurity testing was not conducted on the CRPMS. This decision was based on the absence of personal health information and the fact that the system was hosted on the institution's infrastructure.

## Conclusion

We created a CRPMS and assessed as well as iteratively improved its usability. The findings highlighted the success of our Agile UCD model to create applications that result in high usability and improve user workflow. The methodology of our study can be used as a guide to create similar applications to improve clinical research project management. We will continue working on applying this methodology to further improve the usability and functionality of the CRPMS and expand the scope to other clinical research groups in our institution as well as to other healthcare institutions.

## Clinical Relevance Statement

Effective research project management is crucial in academic hospitals involved in extensive research. The findings of the present study highlight the creation of a research project management application utilizing user-centered design and agile software development that serves as a centralized platform for organizing all ongoing research projects within a hospital, fostering collaboration, and facilitating effective project completion. Usability testing of this application has helped meet the needs of the clinical research staff and the principal investigators. The application has potential to be used by various hospitals to help improve their research project management.

## Multiple-Choice Questions

1. In the formative evaluation of the prototype, which of the following methods was used to measure the usability of the application?
  - Interview of the key users
  - System Usability Scale survey
  - Fibonacci-based scaling
  - Video recording of usability testing
  The correct answer is option b. The System Usability Scale (SUS) is a widely used tool for measuring the usability of a system. The SUS consists of 10 questions, each scored from 1 to 5 (from Strongly Disagreed to Strongly Agreed). The questions cover a range of usability factors, such as ease of use, learnability, efficiency, and overall satisfaction. The SUS score is a sum of all 10 questions multiplied by 2.5, which results in scores ranging from 0 to 100. A system of application with SUS score above 68 is considered to have above-average usability. Options a and d are data collection methods. Option c is for issue prioritization.
2. During the usability evaluation of the prototype system, how were the high-severity issues determined from the total issues that were reported by the participants?
  - By setting an arbitrary cutoff of severity score (>1.5)
  - By dividing the number of users with the number of times an issue was mentioned in each group testing
  - By counting the number of times an issue was mentioned in each group testing
  - By applying Fibonacci-based scaling from 1 to 5
  The correct answer is option a. All the usability issues were reviewed by the development team. Based on the development team feedback and capacity, a cutoff of >1.5 was determined to separate high-severity issues from total issues. Options b, c, and d are the three scoring mechanisms to calculate the severity score.

## Notes

**Erratum:** This article was published with incorrect page numbers. The page numbers have been corrected subsequently with the publication of an erratum (DOI: 10.1055/a-2658-0791).

## References

[1] Findley T W Daum M C Macedo J A Research in physical medicine and rehabilitation. VI. Research project management Am J Phys Med Rehabil 1989 68 06 288 299 2590516 10.1097/00002060-198912000-00006

[2] Lenz E R Strategies for successful research project management Nurs Leadersh Forum 1999 4 01 26 31 10786569

[3] Howe R Flanagan C Case managers getting it done: a project management primer Lippincotts Case Manag 2004 9 03 152 154 15252367 10.1097/00129234-200405000-00008

[4] Nevan Wright J Time and budget: the twin imperatives of a project sponsor Int J Proj Manag 1997 15 03 181 186

[5] Project Management Institute, ed A Guide to the Project Management Body of Knowledge/Project Management Institute 6th ed. Project Management Institute 2017

[6] Clinical Epidemiology and Evidence-Based Medicine Association of Chinese Medical Association [Urgent to implement and perfect clinical research project management regulation] Zhonghua Yi Xue Za Zhi 2019 99 28 2166 2168 31434387 10.3760/cma.j.issn.0376-2491.2019.28.003

[7] Kalankesh L R Nasiry Z Fein R A Damanabi S Factors influencing user satisfaction with information systems: a systematic review Galen Med J 2020 9 e1686 34466567 10.31661/gmj.v9i0.1686 PMC8343607

[8] Holden R J Karsh B T The technology acceptance model: its past and its future in health care J Biomed Inform 2010 43 01 159 172 19615467 10.1016/j.jbi.2009.07.002 PMC2814963

[9] Shneiderman B Designing the User Interface: Strategies for Effective Human-Computer-Interaction 3rd ed. Addison Wesley Longman 1998

[10] Norman D A The Design of Everyday Things. Rev. and expanded edition MIT Press 2013

[11] What is User Centered Design?—updated 2023. IxDF. Accessed December 12, 2023 at:https://www.interaction-design.org/literature/topics/user-centered-design

[12] User Centered Design: definition, benefits, principles, and methodsAccessed December 12, 2023 at:https://uxcam.com/blog/understanding-user-centered-design/

[13] Turning Usability Testing Data into Action. Toptal. Accessed December 12, 2023 at:https://www.toptal.com/designers/usability-testing/turning-usability-testing-data-into-action

[14] Usability testing in design—why is it important? by Shree Harsha. UX Collective. Accessed December 12, 2023 at:https://uxdesign.cc/usability-testing-in-design-and-why-is-it-important-cfddfbbdaac9

[15] What Is Usability Testing?—Baymard Institute. Accessed December 12, 2023 at:https://baymard.com/learn/usability-testing

[16] NEXT. Product discovery platformAccessed December 12, 2023 at:https://www.nextapp.co/glossary/guides/user-centered-design

[17] ISO 9241-11:2018(en), Ergonomics of human-system interaction—Part 11: Usability: Definitions and conceptsAccessed March 11, 2024 at:https://www.iso.org/obp/ui/#iso:std:iso:9241:-11:ed-2:v1:en

[18] User-centered design approach: Complete guide. Opensense Labs. Accessed December 12, 2023 at:https://opensenselabs.com/blog/articles/user-centered-design-approach-core-principles-methods

[19] Making Usability Findings Actionable. Nielsen Norman Group. Accessed February 20, 2025 at:https://www.nngroup.com/articles/actionable-usability-findings/

[20] What is Agile Software Development?Accessed December 12, 2023 at:https://www.visual-paradigm.com/scrum/what-is-agile-software-development/

[21] Adobe Communications Team What is Agile Software Development?. Adobe Workfront. Accessed December 12, 2023 at:https://business.adobe.com/blog/basics/agile-development

[22] What Is Agile Software Development?Aha! Accessed December 12, 2023 at:https://www.aha.io/roadmapping/guide/agile/agile-software-development

[23] What is Scrum?. Scrum.org. Accessed March 26, 2024 at:https://www.scrum.org/resources/what-scrum-module

[24] Peres A L Meira S L Towards a framework that promotes integration between the UX design and SCRUM, Aligned to CMMIIn: 2015 10th Iberian Conference on Information Systems and Technologies (CISTI). IEEE;2015 1 4

[25] Zotov E Hills A F de Mello F L JointCalc: a web-based personalised patient decision support tool for joint replacement Int J Med Inform 2020 142 104217 32853974 10.1016/j.ijmedinf.2020.104217 PMC7607377

[26] Tobias G Spanier A B Developing a mobile app (iGAM) to promote gingival health by professional monitoring of dental selfies: user-centered design approach JMIR Mhealth Uhealth 2020 8 08 e19433 32795985 10.2196/19433 PMC7455872

[27] Melnick E R Lopez K Hess E P Back to the bedside: developing a bedside aid for concussion and brain injury decisions in the emergency department EGEMS (Wash DC) 2015 3 02 1136 26290885 10.13063/2327-9214.1136 PMC4537154

[28] Backman C Harley A Peyton L Development of a path to home mobile app for the geriatric rehabilitation program at bruyère continuing care: protocol for user-centered design and feasibility testing studies JMIR Res Protoc 2018 7 09 e11031 30249591 10.2196/11031 PMC6231760

[29] SMILe study team Leppla L Hobelsberger S Rockstein D Implementation science meets software development to create eHealth components for an integrated care model for allogeneic stem cell transplantation facilitated by eHealth: the SMILe study as an example J Nurs Scholarsh 2021 53 01 35 45 33348461 10.1111/jnu.12621

[30] Barr P J Haslett W Dannenberg M D An audio personal health library of clinic visit recordings for patients and their caregivers (HealthPAL): user-centered design approach J Med Internet Res 2021 23 10 e25512 34677131 10.2196/25512 PMC8727051

[31] Aldossari R Albesher L Alshammari M Challenges of integrating Agile and UX/UCD: systematic literature review 2022 10.13140/RG.2.2.18610.68808

[32] Heart Institute Cincinnati Children'sAccessed December 12, 2023 at:https://www.cincinnatichildrens.org/service/h/heart-institute

[33] Keiser S Vandermar D Garner M B Beyond Design: The Synergy of Apparel Product Development 5th ed. Fairchild Books 2022 10.5040/9781501366581

[34] Affairs AS for P. System Usability Scale (SUS)September 6, 2013. Accessed January 12, 2024 at:https://www.usability.gov/how-to-and-tools/methods/system-usability-scale.html

[35] Maguire M Delahunt B Doing a thematic analysis: a practical, step-by-step guide for learning and teaching scholars AISHE-J Irel J Teach Learn High Educ 2017 9 03 3351 3364

[36] Ferreira J Noble J Biddle R Agile development iterations and UI design. In: AGILE 2007 (AGILE 2007) IEEE 2007 50 58

[37] Losada B Flexible requirement development through user objectives in an Agile-UCD hybrid approachIn: Proceedings of the XIX International Conference on Human Computer Interaction. ACM;2018 1 8

[38] Sensuse D I Satria D Pratama A A Wulandari I A Mishbah M Noprisson H Integrating UCD into Scrumban for better and faster usability designIn: 2017 International Conference on Information Technology Systems and Innovation (ICITSI). IEEE;2017 297 302

[39] Teka D Dittrich Y Kifle M Integrating discount usability in scrum development process in EthiopiaIn: 2017 International Conference on Computing Networking and Informatics (ICCNI). IEEE;2017 1 8

[40] Teka D Dittrich Y Kifle M Adapting lightweight user-centered design with the scrum-based development processIn: Proceedings of the 2018 International Conference on Software Engineering in Africa. ACM;2018 35 42

[41] Williams A User-centered design, activity-centered design, and goal-directed design: a review of three methods for designing web applicationsIn: Proceedings of the 27th ACM International Conference on Design of Communication. ACM;2009 1 8

[42] Ghaben S J Mat Ludin A F Mohamad Ali N Beng Gan K Singh D KA A framework for design and usability testing of telerehabilitation system for adults with chronic diseases: a panoramic scoping reviewDigit Health 2023;9:20552076231191014 10.1177/20552076231191014 PMC10437210 37599901

[43] Qudah B Luetsch K The influence of mobile health applications on patient - healthcare provider relationships: a systematic, narrative review Patient Educ Couns 2019 102 06 1080 1089 30745178 10.1016/j.pec.2019.01.021

[44] Wood R Dixon E Elsayed-Ali S Shokeen E Lazar A Lazar J Investigating best practices for remote summative usability testing with people with mild to moderate dementia ACM Trans Access Comput 2021 14 03 10.1145/3460942 PMC8855365 35186177

[45] Downie A S Hancock M Abdel Shaheed C An electronic clinical decision support system for the management of low back pain in community pharmacy: development and mixed methods feasibility study JMIR Med Inform 2020 8 05 e17203 32390593 10.2196/17203 PMC7248808

[46] Dopp A R Parisi K E Munson S A Lyon A R A glossary of user-centered design strategies for implementation experts Transl Behav Med 2019 9 06 1057 1064 30535343 10.1093/tbm/iby119

[47] Why You Only Need to Test with 5 Users. Nielsen Norman Group. Accessed January 12, 2024 at:https://www.nngroup.com/articles/why-you-only-need-to-test-with-5-users/

