# Erratum: Agile User-Centered Design of a Clinical Research Project Management System in a Pediatric Health Institute

**DOI:** 10.1055/a-2658-0791

**Published:** 2025-08-04

**Authors:** Danny T.Y. Wu, Manhar Suharwardy, Aniruddhan Ramesh, Shubhra Gupta, Chen Xin, Ting Zhang, Ching-Tzu Tsai, Jenna Dawn, Michelle Faust, Adam Kushner

**Affiliations:** 1 Department of Biostatistics, Health Informatics and Data Sciences, University of Cincinnati College of Medicine, Ohio, United States; 2 Department of Pediatrics, University of Cincinnati College of Medicine, Cincinnati, Ohio, United States; 3 Medical Sciences Baccalaureate Program, University of Cincinnati College of Medicine, Ohio, United States; 4 School of Design, College of Design, Architecture, Art, and Planning, University of Cincinnati, Ohio, United States; 5 Department of Computer Science, College of Engineering and Applied Sciences, University of Cincinnati, Ohio, United States; 6 Heart Institute, Cincinnati Children's Hospital Medical Center, Cincinnati, Ohio, United States; 7 School of Information and Library Science, University of North Carolina at Chapel Hill, North Carolina, United States

The above article published in *Applied Clinical Informatics Open* , Volume 9, Number 2, e1-e11, on July 9, 2025 (doi:  ) was published with incorrect page numbers. The page numbers have been corrected subsequently with the publication of this erratum.

