# Supplementary material for: Agile User-Centered Design of a Clinical Research Project Management System in a Pediatric Health Institute
Source: ACI open. 2025 Jul 9;9(2):e30–40. doi: 10.1055/a-2625-1046 (PMC13393553; doi:10.1055/a-2625-1046)
Supplement: Supplementary file 1 — Supplementary Material [file 10-1055-a-2625-1046_26519142.pdf]

Supplementary Appendix 1 System Usability Scale

		1<br>Strongly disagree	2<br>Disagree	3<br>Neutral	4<br>Agree	5<br>Strongly agree
1	I think that I would like to use this system frequently					
2	I found the system unnecessarily complex					
3	I thought the system was easy to use					
4	I think that I would need the support of a technical person to be able to use this system					
5	I found the various functions in this system were well integrated					
6	I thought there was too much inconsistency in this system					
7	I would imagine that most people would learn to use this system very quickly					
8	I found the system very awkward to use					
9	I felt very confident using the system					
10	I needed to learn a lot of things before I could get along with this system					

Notes: The System Usability Scale (SUS) is calculated based on responses to 10 questions, each rated on a 5-point Likert scale (1 = Strongly Disagree, 5 = Strongly Agree). To compute the score, responses to odd-numbered questions (1, 3, 5, etc.) are adjusted by subtracting 1, whereas responses to even-numbered questions (2, 4, 6, etc.) are adjusted by subtracting them from 5. The adjusted values are then summed, and the total is multiplied by 2.5, resulting in a final SUS score ranging from 0 to 100 (though not a percentage). Generally, a SUS score above 68 indicates above-average usability, while lower scores suggest usability concerns.

Supplementary Appendix 2 Severity Score

The **Severity Score** of a usability issue was determined by multiplying the score of Impact, Task Criticality, and Frequency. **Impact** was rated on a Fibonacci-based scale from 1, 2, 3, and 5, where 5 indicated a blocker preventing task completion, 3 represented a major issue causing frustration or delays, 2 was a minor issue affecting task performance, and 1 was a participant suggestion. **Task Criticality** was rated from 1 to 3, with 3 signifying a necessary task for workflow completion, 2 indicating an important but non-essential task, and 1 representing a task with minimal impact on the overall workflow. **Frequency** was calculated by dividing the number of users affected by the number of times the issue occurred across test groups. The severity scores of all issue were normalized and ranked.

References

13 Turning Usability Testing Data into Action. Toptal. Accessed December 12, 2023 at: https://www.toptal.com/designers/us-ability-testing/turning-usability-testing-data-into-action
19 Making Usability Findings Actionable. Nielsen Norman Group. Accessed February 20, 2025 at: https://www.nngroup.com/articles/actionable-usability-findings/

 © 2025. The Author(s).
